# Omega-3 polyunsaturated fatty acid attenuates the inflammatory response by modulating microglia polarization through SIRT1-mediated deacetylation of the HMGB1/NF-κB pathway following experimental traumatic brain injury

**DOI:** 10.1186/s12974-018-1151-3

**Published:** 2018-04-20

**Authors:** Xiangrong Chen, Chunnuan Chen, Sining Fan, Shukai Wu, Fuxing Yang, Zhongning Fang, Huangde Fu, Yasong Li

**Affiliations:** 1 The Second clinical medical college, The Second Affiliated Hospital, Fujian Medical University, Quanzhou, 362000 Fujian Province China; 2 Department of Neurosurgery, Affiliated Hospital of YouJiang Medical University for Nationalities, Baise, 533000 Guangxi Province China

**Keywords:** Traumatic brain injury, Omega-3 polyunsaturated fatty acid, Microglia polarization, Neuroinflammation, Sirtuin1, HMGB1/NF-κB pathway

## Abstract

**Background:**

Microglial polarization and the subsequent neuroinflammatory response are contributing factors for traumatic brain injury (TBI)-induced secondary injury. High mobile group box 1 (HMGB1) mediates the activation of the NF-κB pathway, and it is considered to be pivotal in the late neuroinflammatory response. Activation of the HMGB1/NF-κB pathway is closely related to HMGB1 acetylation, which is regulated by the sirtuin (SIRT) family of proteins. Omega-3 polyunsaturated fatty acids (ω-3 PUFA) are known to have antioxidative and anti-inflammatory effects. We previously demonstrated that ω-3 PUFA inhibited TBI-induced microglial activation and the subsequent neuroinflammatory response by regulating the HMGB1/NF-κB signaling pathway. However, no studies have elucidated if ω-3 PUFA affects the HMGB1/NF-κB pathway in a HMGB1 deacetylation of dependent SIRT1 manner, thus regulating microglial polarization and the subsequent neuroinflammatory response.

**Methods:**

The Feeney DM TBI model was adopted to induce brain injury in rats. Modified neurological severity scores, rotarod test, brain water content, and Nissl staining were employed to determine the neuroprotective effects of ω-3 PUFA supplementation. Assessment of microglia polarization and pro-inflammatory markers, such as tumor necrosis factor (TNF)-α, interleukin (IL)-1β, IL-6, and HMGB1, were used to evaluate the neuroinflammatory responses and the anti-inflammatory effects of ω-3 PUFA supplementation. Immunofluorescent staining and western blot analysis were used to detect HMGB1 nuclear translocation, secretion, and HMGB1/NF-κB signaling pathway activation to evaluate the effects of ω-3 PUFA supplementation. The impact of SIRT1 deacetylase activity on HMGB1 acetylation and the interaction between HMGB1 and SIRT1 were assessed to evaluate anti-inflammation effects of ω-3 PUFAs, and also, whether these effects were dependent on a SIRT1-HMGB1/NF-κB axis to gain further insight into the mechanisms underlying the development of the neuroinflammatory response after TBI.

**Results:**

The results of our study showed that ω-3 PUFA supplementation promoted a shift from the M1 microglial phenotype to the M2 microglial phenotype and inhibited microglial activation, thus reducing TBI-induced inflammatory factors. In addition, ω-3 PUFA-mediated downregulation of HMGB1 acetylation and its extracellular secretion was found to be likely due to increased SIRT1 activity. We also found that treatment with ω-3 PUFA inhibited HMGB1 acetylation and induced direct interactions between SIRT1 and HMGB1 by elevating SIRT1 activity following TBI. These events lead to inhibition of HMGB1 nucleocytoplasmic translocation/extracellular secretion and alleviated HMGB1-mediated activation of the NF-κB pathway following TBI-induced microglial activation, thus inhibiting the subsequent inflammatory response.

**Conclusions:**

The results of this study suggest that ω-3 PUFA supplementation attenuates the inflammatory response by modulating microglial polarization through SIRT1-mediated deacetylation of the HMGB1/NF-κB pathway, leading to neuroprotective effects following experimental traumatic brain injury.

## Background

Neuronal inflammation induced by activation of microglia is a vital contributing factor of traumatic brain injury (TBI)-induced secondary injury [1–3]. After trauma, resident microglia and peripheral macrophages migrate to the site of injury and secrete a large number of inflammatory cytokines, such as tumor necrosis factor (TNF), interleukins (IL), and interferons (IFN) that cause acascade of inflammatory responses and neuronal apotosis [4, 5]. Transition of microglia from the anti-inflammatory (M2) to the pro-inflammatory (M1) phenotype play a crucial role in microglial activation and the subsequent neuroinflammatory response. The M1 phenotype favors pro-inflammatory cytokine release that exacerbates neural damage while the M2 phenotype promotes neurotrophic factors that contribute to neural repair [6–8]. Wang et al. [6] found that microglia respond to TBI with a transient M2 phenotype, followed by a transition to M1, which is strongly correlated with the severity of TBI. Thus, inhibition of microglial dysfunction and its phenotypic switch to the M1 phenotype may offer new anti-inflammatory strategies to improve the recovery in TBI patients [2, 9].

High mobile group box 1 (HMGB1) is considered to be the central component of the late inflammatory response [10, 11]. Translocation and secretion of HMGB1 are important steps in HMGB1-induced inflammation [12]. After release, extracellular HMGB1 activates several cell surface receptors including advanced glycation end products, toll-like receptors, and chemokine (C-X-C motif) receptor 4, through both medullary differentiation factor (MyD88) and non-MyD88-dependent pathways. These pathways then trigger a signal cascade, either directly through the nuclear factor-κB (NF-κB) pathway or indirectly via the phosphatidylinositol 3-kinase or mitogen-activated protein kinase pathway [13–16]. Following nuclear translocation of NF-κB, cells release large numbers of inflammatory cytokines initiating a cascade amplification of inflammatory responses. Activation of the NF-κB pathway is associated with HMGB1 expression and the post-TBI inflammatory response [17, 18]. Our previous study [4] confirmed that inhibiting HMGB1 translocation and release, and also HMGB1-mediated activation of the NF-κB signaling pathway, led to a reduction in TBI-induced microglial activation and the subsequent inflammatory response, thus providing neuroprotection following TBI.

Activation of the HMGB1/NF-κB pathway is influenced by post-translational modifications, including acetylation levels, which are regulated by the balanced expression of histone deacetylases (HDACs) and histone acetyltransferases (HATs) [19, 20]. These in turn regulate HMGB1 intranuclear translocation and mobility by a lysine acetylation dependent mechanism. Thus, hyperacetylation of HMGB1 is also known to affect its DNA binding activity and intranuclear translocation [21]. The sirtuin (SIRT) family of proteins are a family of nicotinamide adenine dinucleotide (NAD+)-dependent class III HDACs, which include SIRT1through SIRT7 [22, 23]. Among the SIRT family, SIRT1 is one of the most studied deacetylases and has been shown to regulate the inflammatory response through deacetylation of lysine in HMGB1 which HMGB1 suppresses transcription [24–26]. Kim et al. [27] recently showed that SIRT1 promoted the deacetylation of HMGB1 and thus inhibited HMGB1 transcription and extracellular release in LPS-activated macrophages. These findings suggest that HMGB1 may be a novel deacetylation target of SIRT1, which in turn inhibits the HMGB1/NF-κB pathway through HMGB1 deacetylation, although, to date, the mechanism remains unclear.

Omega-3 polyunsaturated fatty acids (ω-3 PUFA) include eicosapentaenoic acid and docosahexaenoic acid, which are known to be biologically active compounds with antioxidative and anti-inflammatory effects, all of which influence the pathogenesis of many diseases, including Alzheimer’s disease [28], acute pancreatitis [29], Parkinson’s disease [30], and cerebral ischemia [31]. Accumulating evidence has demonstrated that ω-3 PUFA inhibits TBI-induced inflammatory responses and that this inhibitory mechanism may be related to microglial activation [32–34]. We previously reported that ω-3 PUFA supplementation inhibited TBI-induced microglial activation and the subsequent inflammatory response by regulating the HMGB1/NF-κB signaling pathway, which lead to neuroprotective effects [4]. In addition, we also found that SIRT1 levels were upregulated after ω-3 PUFA supplementation, indicating that ω-3 PUFA inhibited the expression, translocation, and release of HMGB1 in a SIRT1 deacetylation-mediated dependent manner [4]. To date, no studies have elucidated if ω-3 PUFA affects the HMGB1/NF-κB pathway through a HMGB1 deacetylation-dependent SIRT1 mechanism in a TBI-induced microglial activation model. Thus, in this present study, the neuroprotective effects of ω-3 PUFAs against TBI-induced inflammation were studied. In addition, the potential molecular mechanisms focusing on the phenotypic transition of microglia and the SIRT1-mediated HMGB1/NF-κB deacetylation were also investigated.

## Methods

### Animals

All animal experiments were approved by the Fujian Provincial Medical University Experimental Animal Ethics Committee (Fuzhou, China) and were performed under strict supervision. Adult male Sprague-Dawley rats, ranging between 230 and 260 g, were purchased from the Experimental Animal Facility in Fujian Medical University and housed in a temperature (23 ± 2 °C) and light (12-h light/dark cycle) controlled room with ad libitum access to food and water.

### Experimental model and drug administration

All rats were randomly assigned into a sham group, sham + ω-3 PUFA supplementation group (sham + ω-3 group), TBI group, and TBI + ω-3 group. After injury, the groups were further divided into four subgroups: a 1 day group, a 3 day group, and a 7 day and 14 day group (*n* = 12 each). Six rats in each group were sacrificed for neurological evaluation and histological studies; the remaining six rats were used for molecular studies. TBI was induced in anesthetized (50 mg/kg sodium pentobarbital; intraperitoneally) rats as described previously [4]. Briefly, a midline incision was made over the skull, and a 5-mm craniotomy was drilled through the skull 2 mm caudal to the left coronal suture and 2 mm from the midline without disturbing the dura. TBI was induced using a weight-drop hitting device (ZH-ZYQ, Electronic Technology Development Co., Xuzhou, China) with a 4.5-mm-diameter cylinder bar weighing 40 g from a height of 20 cm. Bone wax was used to seal the hole, and the scalp was sutured. All procedures were the same for each group except in the sham group, in which no weight was dropped. Approximately 30 min after TBI, the TBI + ω-3 group was intraperitoneally injected with ω-3 PUFA (2 ml/kg; Sigma, St. Louis, MO, USA) once per day for 7 consecutive days [4]. To inhibit SIRT1 signaling, 30 ul/kg Sirtinol (2 mmol/l, diluted indimethyl sulfoxide) was administrated into the left lateral ventricle 24 h after intraperitoneal ω-3 PUFA injection, to clarify the role of SIRT1 in ω-3 PUFA-mediated neuroprotection [22]. The remaining groups were injected with same dose of vehicle as a control.

### Measurement of neurological impairment score and rotarod test

Neurological deficit was calculated using neurological impairment score. Rats were subjected to exercise (muscular state and abnormal action), sensation (visual, tactile, and balance), and reflex examinations and assigned a modified neurological severity score (mNSS) [4] that was recorded when a task failed to be completed or when the corresponding reflex was lost. The mNSS test was graded on a scale of 0–18, where a total score of 18 points indicated severe neurological deficits and a score of 0 indicated normal performance, 13–18 points indicated severe injury, 7–12 indicated mean-moderate injury, and 1–6 indicated mild injury. Neurological function was measured at different time points by investigators who were blinded to group information.

The rotarod protocol was modified slightly from that in the previous report [35]. Briefly, rats underwent a 2-day testing phase with rotarod (IITC Life Science, Woodland Hills, CA, USA), which gradually accelerated from 5 to 45 rpm over 5 min. During the procedure, the latency to fall was recorded as the time before rats fell off the rod or gripped around for two successive revolutions from day 3 after TBI. The mean latency was measured at different time points by investigators who were blinded to group information.

### Measurement of brain water content and blood-brain barrier (BBB) permeability

Brain water content was calculated using the wet weight-dry weight method [4]. Animals were sacrificed after the mNSS test, and their cortices were removed at the edge of the bone window (200 ± 20 mg). Filter paper was used to remove excess blood and cerebrospinal fluid. The wet weight was measured, and the brains were dried in an oven at 100 °C for 24 h until a constant weight was achieved, at which point the dry weight was measured. The % brain water content was calculated as: (wet weight − dry weight)/wet weight × 100%.

BBB permeability was investigated by measuring the extravasation of Evans blue (dye) (Sigma Aldrich) [36]. Evans blue (2% in saline; 4 mL/kg) was intravenously injected 2 h prior to sacrifice 3 days after TBI. Following sacrifice, mice were transcardially perfused with PBS followed by PBS containing 4% paraformaldehyde. Each tissue sample was immediately weighed and homogenized in a solution containing 1 mL 50% trichloroacetic acid. The samples were then centrifuged and the absorption of the supernatant was measured by a spectrophotometer (UV-1800 ENG 240V; Shimadzu Corporation, Japan) at a wave length of 620 nm. The quantity of Evans blue was calculated using a standard curve and expressed as micrograms of Evans blue/g of brain tissue using a standardized curve.

### Nissl staining

Formaldehyde-fixed specimens were embedded in paraffin and cut into 4-μm-thick sections that were deparaffinized with xylene and rehydrated in a graded series of alcohol. Samples were treated with Nissl staining solution for 5 min. Damaged neurons were shrunken or contained vacuoles, whereas normal neurons had a relatively large, full soma and round, large nuclei. Average intensities or cell counts were calculated from the same sections in six rats per group with Image Pro Plus 7.0 by investigators who were blinded to the experimental groups.

### Immunohistochemical analysis

Formaldehyde-fixed specimens were embedded in paraffin and cut into 4-μm-thick sections that were deparaffinized with xylene and rehydrated in a graded series of alcohol. Antigen retrieval was carried out by microwaving in citric acid buffer. Sections were incubated with an antibody against SIRT1 (1:100; Cell Signaling Technology, Danvers, MA, USA), washed, and then incubated with secondary antibody for 1 h at room temperature. The negative control was prepared without the addition of the anti-SIRT1 antibody. A total of five sections from each animal were used for quantification, and the signal intensity was evaluated as follows [4]: 0, no positive cells; 1, very few positive cells; 2, moderate number of positive cells; 3, large number of positive cells; and 4, the highest number of positive cells.

### Immunofluorescence analysis

Formaldehyde-fixed specimens were embedded in paraffin and cut into 4-μm-thick sections that were deparaffinized with xylene and rehydrated in a graded series of alcohol, followed by antigen retrieval. Sections were incubated overnight at 4 °C with antibodies against CD16 (1:200, Abcam, Cambridge, UK), CD206 (1:200; Abcam), Neuronal nuclei (1:100; Boster Biotech, Wuhan, China), ionized calcium-binding adapter molecule (Iba)-1 (1:200; Santa Cruz Biotechnology, Santa Cruz, CA, USA), GFAP (1:200; Abcam), and HMGB1 (1:100; Cell Signaling Technology). After washing, the sections were incubated with secondary antibodies for 1 h at room temperature. Cell nuclei were stained with 4′,6-diamidino-2-phenylindole. Immunopositive cells in five selected fields were counted under a microscope (Leica, Wetzlar, Germany) at × 400 magnification by investigators who were blinded to the experimental groups.

### Terminal deoxynucleotidyl transferase dUTP nick-end labeling (TUNEL) assay

Apoptotic cells were detected using a TUNEL kit (Roche Diagnostics, Indianapolis, IN, USA) according to the manufacturer’s instructions. Indicators of apoptosis included a shrunken cell body, irregular shape, nuclear condensation, and brown diaminobenzidine staining, as observed by microscopy at × 400 magnification. The final average percentage of TUNEL-positive cells of the six sections was regarded as the data for each sample.

### Enzyme-linked immunosorbent assay (ELISA)

Inflammatory factors in brain tissue were detected using ELISA kits for TNF-α, IL-1β, IL-6, HMGB1, and IL-10 (all from Boster Biotech, Wuhan, China). Measured OD values were converted into a concentration value.

### Western blotting

Proteins were extracted with radioimmunoprecipitation assay lysis buffer (sc-24948; Santa Cruz Biotechnology). Proteins (30 μg) were separated by sodium dodecyl sulfate-polyacrylamide gel electrophoresis and transferred to a polyvinylidene difluoride membrane that was probed with primary antibodies against B cell lymphoma (Bcl)-2 (1:400), Bcl-2-associated X factor (Bax) (1:200), CD16 (1:200), CD206 (1:200); and GFAP (1:400) (all from Abcam); and cleaved caspase-3 (1:200), Iba-1 (1:100), and NF-κB p65 (1:200) (all from Cell Signaling Technology), followed by incubation with appropriate secondary antibodies. Immunoreactivity was visualized with the ECL Western Blotting Detection System (Millipore, Billerica, MA, USA). Gray value analysis was conducted with the UN-Scan-It 6.1 software (Silk Scientific Inc., Orem, UT, USA). Expression levels were normalized against β-actin (1:5000, Boster Biotech) or laminin B1 (1:3000, Cell Signaling Technology).

### Co-immunoprecipitation

Brain tissue from lesioned cortices were incubated with 1 μg of SIRT1 (Cell Signaling Technology) or HMGB1 anti-acetylated lysine antibody (Cell Signaling Technology) for 2 h at 4 °C. A 10-μl volume of protein A/G agarose beads (Roche, Mannheim, Germany) was added to the samples and incubated overnight. After immunoprecipitation and centrifugation, agarose beads were washed three times with lysis buffer, and the degree of acetylation of SIRT1 or HMGB1 was analyzed by western blotting using an anti-acetylated lysine antibody (Cell Signaling Technology).

### SIRT1 deacetylase activity and NF-κB DNA binding activity assay

Nucleoproteins were extracted, and their concentrations were determined using the bicinchoninic acid assay. The SIRT1/SIR2 Deacetylase Fluorometric Assay Kit (Cyclex, Nagano, Japan) was used to detect HDAC activity by measuring the absorbance at 460 nm on a microplate reader (2030 ARVO; PerkinElmer LifeSciences, Boston, MA, USA) according to the manufacturer’s instructions. A transcription factor binding assay colorimetric ELISA kit (Cayman Chemical, Ann Arbor, MI) was used to detect NF-κB p65 DNA binding activity by measuring the absorbance at 450 nm on a microplate reader (2030 ARVO).

### NAD+/NADH Quantification Colorimetric Kit

The NAD+/NADH ratio was measured using the NAD+/NADH Quantification Kit (Yusen Biotech, Shanghai, China) according to the manufacturer’s instructions. The absorbance at 450 nm of the mixture was measured by a microplate reader (2030 ARVO).

### Statistical analysis

All statistical analyses were performed using SPSS 18.0 statistical software (SPSS Inc., Chicago, IL, USA). The results were expressed as mean ± standard deviation. Statistical differences among the groups were assessed by one-way ANOVA, and post hoc multiple comparisons were performed using Student-Newman-Keuls tests. Values of *p* < 0.05 were considered statistically significant.

## Results

### Neuroprotective effects of ω-3 PUFA supplementation on TBI

The average blood pressure was lower during anesthesia with sodium pentobarbital than the baseline values throughout the sham group and sham + ω-3 group. However, there were no significant differences in the average blood pressure and arterial blood gas among the TBI groups (data not shown).

**Fig. 1:**
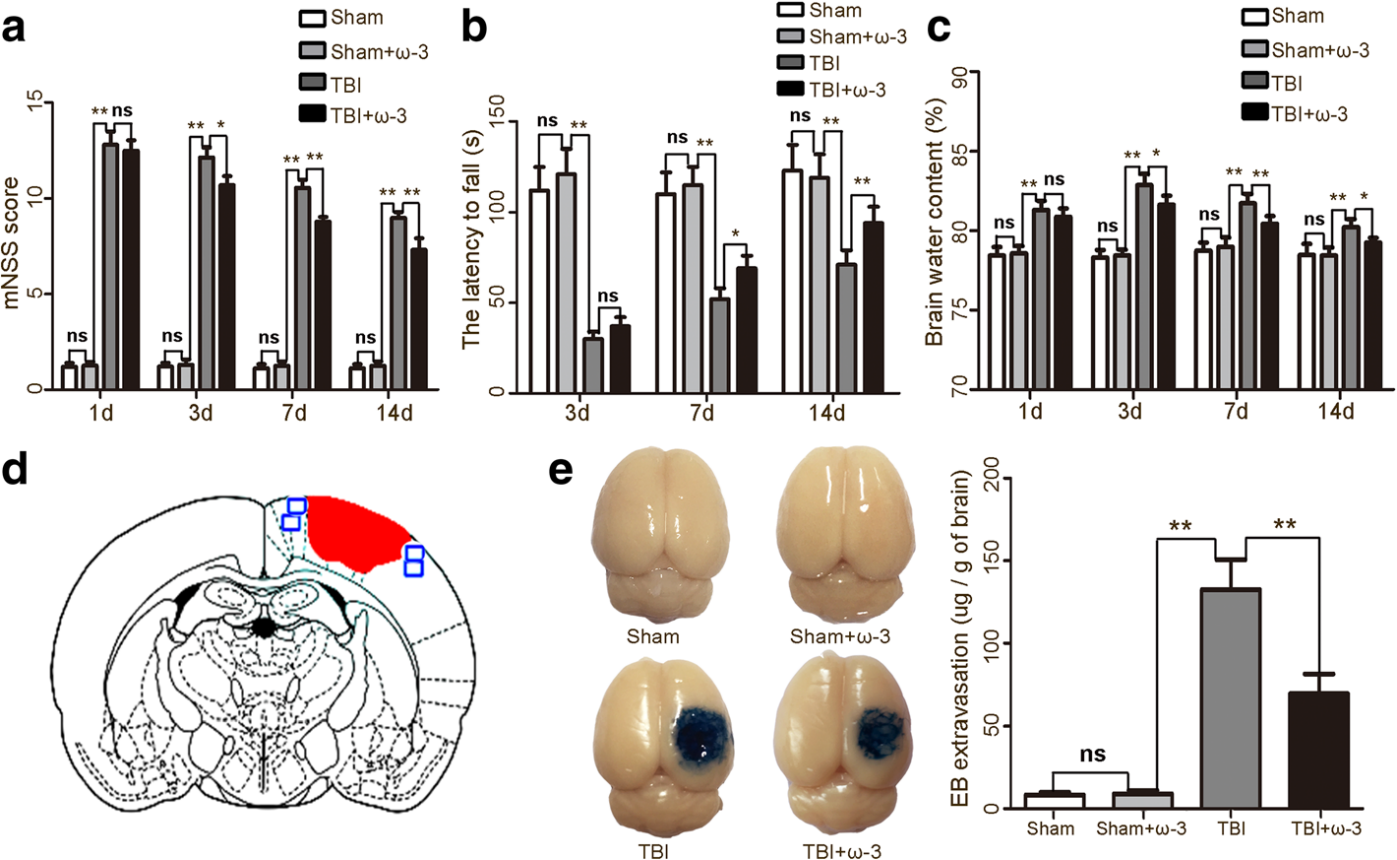
ω-3 PUFA supplementation improves neurological function, brain edema, and BBB permeability after TBI. **a** ω-3 PUFA supplementation improved neurological functions 3 days after TBI (10.69 ± 0.48 vs. 12.14 ± 0.52, *p* < 0.05). **b** Rats in the TBI + ω-3 PUFA group showed significantly improved rotarod performances than rats in the TBI groups from day 7 after TBI. **c** ω-3 PUFA supplementation decreased brain water content 3 days after TBI (81.54 ± 0.57% vs. 82.87 ± 0.73%, *p* < 0.05). **d** A schematic of a brain section after TBI. Areas in red refer to lesioned sites and areas in blue refer to sample points. **e** The TBI group had more Evans blue dye extravasation in the cortex 3 days after TBI compared with the sham group (*p* < 0.05). Compared with the TBI group, the TBI + ω-3 PUFA group had significantly decreased Evans blue dye extravasation of (*p* < 0.05). Representative photos of Evans blue dye extravasation in the experimental groups. Values are expressed as mean ± standard deviation (*n* = 6 per group). N.S., *p* > 0.05, *p < 0.05, **p < 0.01

The neurological function scores of the sham and sham + ω-3 PUFA groups were unaltered at the corresponding time points (scored 1–3). However, neurological function was severely impaired 1 day after TBI (12.78 ± 0.69); from day 3 after TBI, rats in the TBI + ω-3 PUFA group showed significantly better neurological functions than rats in the TBI groups (10.69 ± 0.48 vs.12.14 ± 0.52, *p* < 0.05) (Fig. 1a). Meanwhile, rats in the TBI + ω-3 PUFA group showed significantly improved rotarod performances than rats in the TBI groups from day 7 after TBI (Fig. 1b).

Brain water content is an important predictor of TBI prognosis [4]. Compared with sham group, the water content of brain tissue was higher (82.87%) in the TBI group 3 days after injury (*p* < 0.05). The water content of the TBI + ω-3 PUFA group was markedly lower than that of TBI group (81.54 ± 0.57% vs. 82.87 ± 0.73%, *p* < 0.05) (Fig. 1c).

BBB permeability was investigated by measuring the extravasation of Evans blue [37]. Results demonstrated that the TBI group had more Evans blue dye extravasation in the cortex 3 days after TBI compared to the sham and sham + ω-3 PUFA groups, respectively (*p* < 0.05 for each). Interestingly, compared to the TBI group, the TBI + ω-3 PUFA group had significantly decreased of Evans blue dye extravasation (*p* < 0.05) (Fig. 1e).

### ω-3 PUFA supplementation protects neurons against TBI-induced neuronal apoptosis

**Fig. 2:**
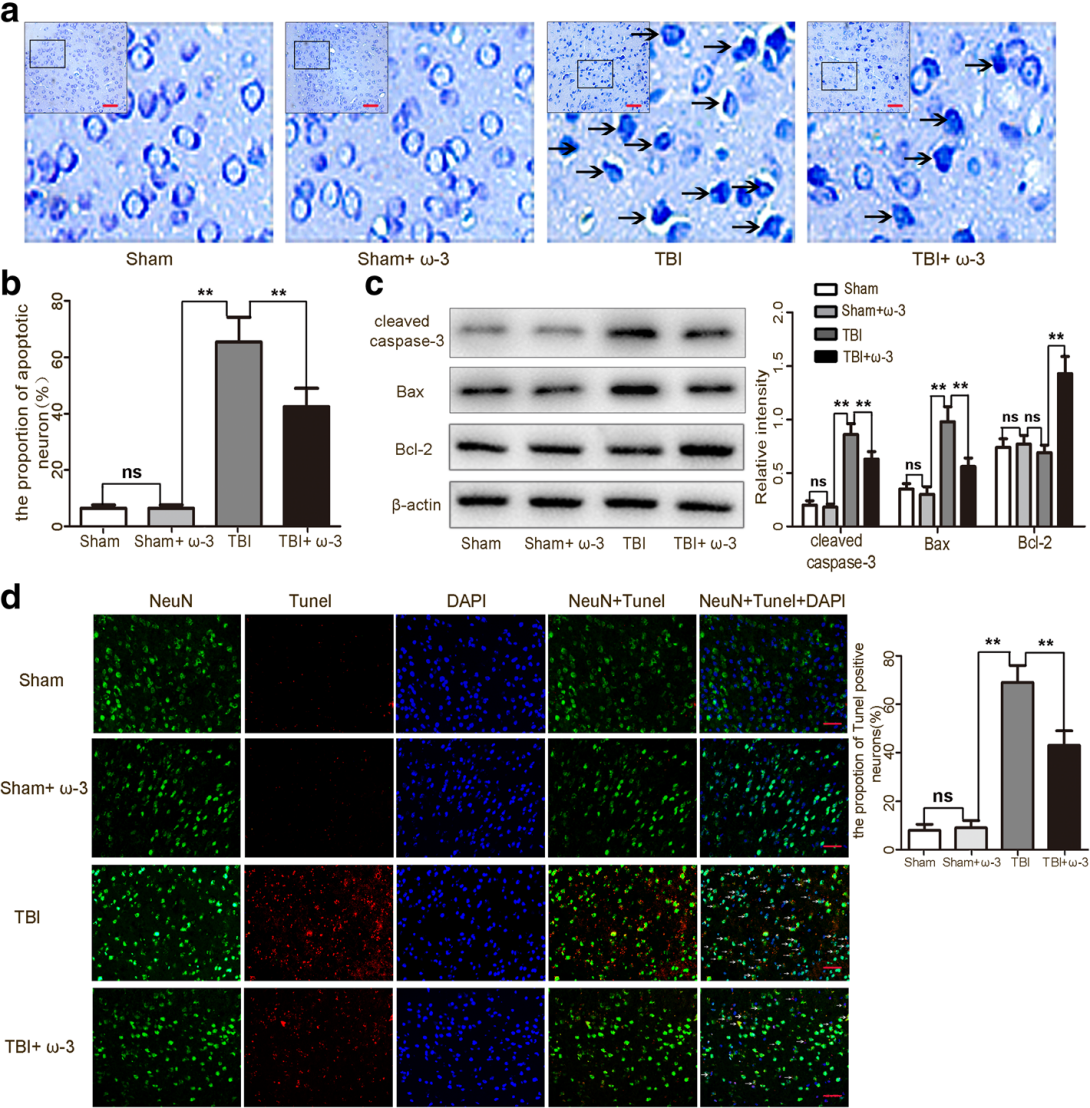
ω-3 PUFA supplementation protects neurons against TBI-induced neuronal apoptosis in the lesioned cortex 3 day after TBI. **a**, **b** The sham group and the sham + ω-3 PUFA group had very low fractions of apoptotic neurons. The percentage of apoptotic cells was higher in the TBI group than in the sham group (*p* < 0.05); the apoptotic fraction was significantly lower in the TBI + ω-3 PUFA group than in the TBI group (42.49 ± 6.53% vs. 66.23 ± 8.46%, *p* < 0.05). Representative photomicrographs of Nissl-stained neurons are shown; arrows indicate apoptotic neurons. **c** Western blot analyses revealed that TBI resulted in the upregulation of apoptotic factors in the cortex; however, compared with the TBI group, cleaved caspase-3 and Bax levels were decreased, whereas the anti-apoptotic factor, Bcl-2, was increased in TBI + ω-3 PUFA group (*p* < 0.05). **d** TUNEL staining demonstrated that TUNEL-positive neurons were significantly decreased in the TBI + ω-3 group compared with the TBI group (43.32 ± 6.03% vs. 69.03 ± 7.31%, *p* < 0.05). Representative photomicrographs of TUNEL-positive neurons are shown; arrows indicate apoptotic neurons. Values are expressed as mean ± standard deviation (*n* = 6 per group). N.S., *p* > 0.05, *p < 0.05, **p < 0.01. Scale bars = 50 μm

Nissl staining was used to identify apoptotic neurons in lesioned cortices [4]. The sham group and the sham + ω-3 PUFA group showed a very low apoptotic fraction of neurons. The percentage of apoptotic cells was higher in the TBI group than in the sham group 3 days after TBI (*p* < 0.05), while the apoptotic fraction was significantly lower in the TBI + ω-3 PUFA than in the TBI group (42.49 ± 6.53% vs.66.23 ± 8.46%, *p* < 0.05) (Fig. 2a, b). Western blot analyses revealed that TBI resulted in the upregulation of apoptotic factors in the cortex 3 days after TBI; however, compared to the TBI group, cleaved caspase-3 and Bax levels were decreased, whereas the anti-apoptotic factor, Bcl-2, was increased in the TBI + ω-3 PUFA group (*p* < 0.05) (Fig. 2c). TUNEL staining further demonstrated that TUNEL-positive neurons were significantly decreased in the TBI + ω-3 group 3 days after TBI compared the TBI group (43.32 ± 6.03% vs.69.03 ± 7.31%, *p* < 0.05) (Fig. 2d). These results suggest that ω-3 PUFA supplementation has no obvious effect on the normal cortex, while it exerts a neuroprotective effect in the lesioned cortex.

### ω-3 PUFA supplementation promotes M2 microglia polarization and alleviates the microglia-mediated inflammatory response

**Fig. 3:**
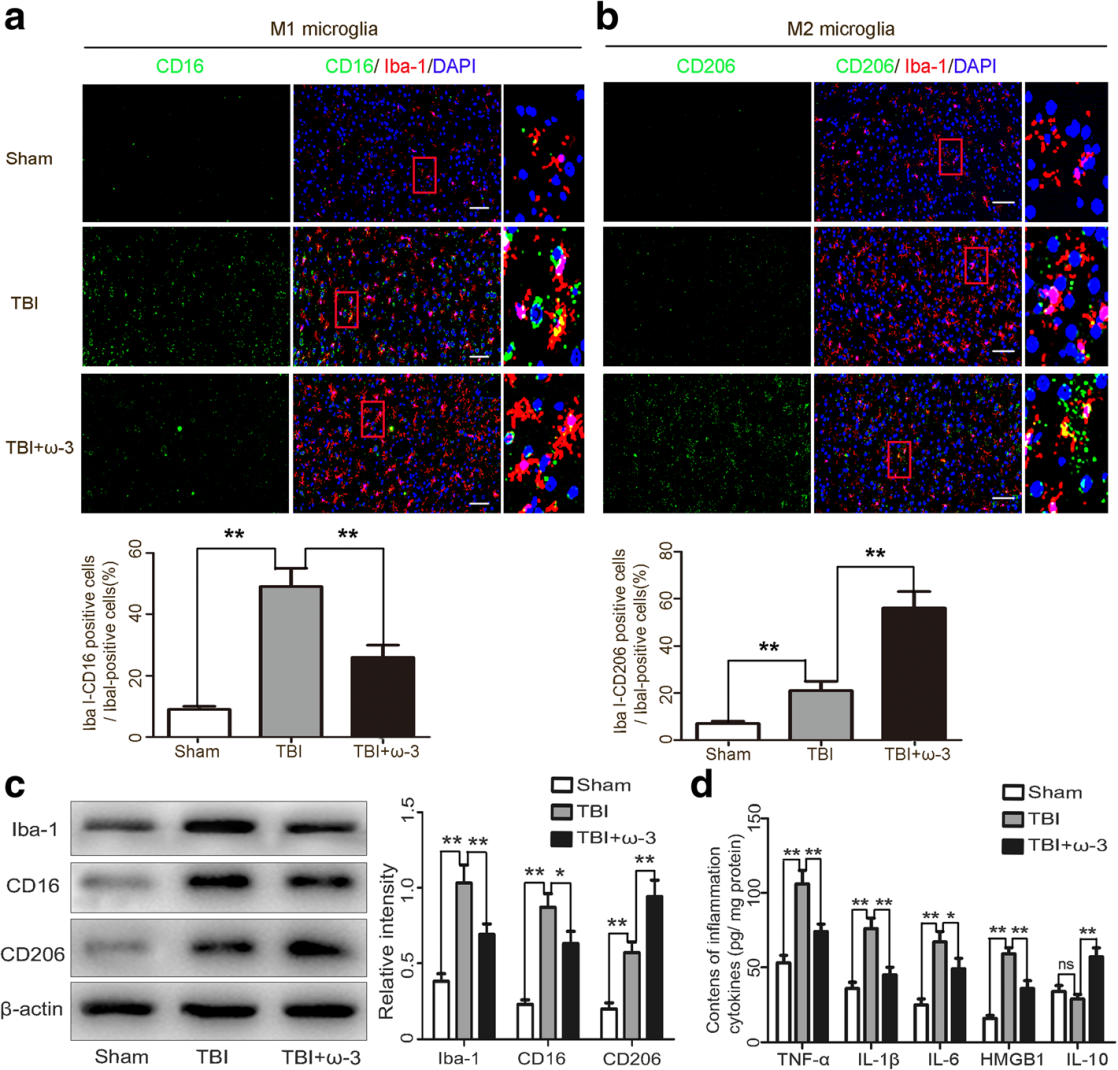
ω-3 PUFA supplementation promotes microglia polarization toward M2 and alleviatesmicroglial-mediated inflammatory responses. **a**, **b** Double staining was used to assess microglia (Iba1+) and a M1-associated marker (CD16+) or a M2-associated marker (CD206+) in the lesioned cortex 3 days after TBI. CD16+-positive microglia increased after TBI but significantly decreased following ω-3 PUFA supplementation, whereas CD206+-positive microglia increased following ω-3 PUFA supplementation. Representative photomicrographs of CD16- or CD206-positive microglias are shown. **c** Western blot analysis showed that CD16 was significantly inhibited, but CD206 was significantly increased after ω-3 PUFA supplementation. **d** ω-3 PUFA supplementation significantly decreased TBI-induced enhancement of TNF-α, IL-1β, IL-6, and HMGB1, while increased the expression of anti-inflammatory IL-10 (*p* < 0.05). Values are expressed as mean ± standard deviation (*n* = 6 per group). N.S., *p* > 0.05, *p < 0.05, **p < 0.01. Scale bars = 50 μm

A microglial transition from the anti-inflammatory (M2) phenotype to the pro-inflammatory (M1) phenotype plays a crucial role both in the microglial activation and the subsequent neuroinflammatory response [1, 2]. To test for shifts in microglial polarization, we double-stained microglia with a pan microglial marker, Iba-1, and the M1-associated marker, CD16, or the M2-associated marker, CD206, 3 days after TBI. As expected, microglia labeled with the M1-associated marker (CD16+) were increased after TBI but significantly decreased by ω-3 PUFA supplementation, whereas the M2-associated marker (CD206+) increased after ω-3 PUFA supplementation. Therefore, ω-3 PUFA supplementation promoted a shift of microglia from the M1 phenotype to the M2 phenotype (Fig. 3a, b). Moreover, western blot analysis showed that CD16 was significantly inhibited, whereas CD206 was significantly increased after ω-3 PUFA supplementation (Fig. 3c). Expression levels of inflammatory factors (TNF-α, IL-1β, IL-6, and HMGB1) were measured after TBI using an ELISA kit, and results also showed that the TBI group had significantly higher expression levels of inflammatory factors compared with the sham group, while ω-3 PUFA supplementation decreased TBI-induced expression of these factors, and increased the expression of anti-inflammatory IL-10 (*p* < 0.05) (Fig. 3d). These findings suggest that ω-3 PUFA supplementation shifts microglia polarization toward the M2 phenotype and alleviates the microglial-mediated inflammatory response.

### ω-3 PUFA supplementation inhibits HMGB1/NF-κB pathway in lesioned cortices

**Fig. 4:**
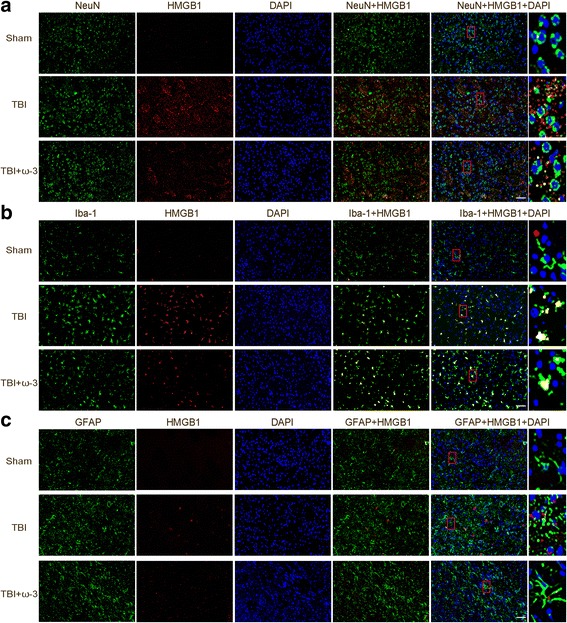
ω-3 PUFA supplementation inhibits HMGB1 expression in lesioned cortices 3 days after TBI. Double staining was used to assessneurons (NeuN+), microglia (Iba-1+), and astrocytes (GFAP+) in the lesioned cortex. **a** TBI enhanced the expression of HMGB1 in neurons, which was significantly decreased by ω-3 PUFA supplementation. Representative photomicrographs of HMGB1-positive neurons are shown. **b** TBI enhanced the expression of HMGB1 in microglia, which was significantly decreased by ω-3 PUFA supplementation. Representative photomicrographs of HMGB1-positive microglias are shown. **c** ω-3 PUFA supplementation had no obvious effect on the expression of HMGB1 in astrocytes. Representative photomicrographs of HMGB1-positive astrocytes are shown. Scale bars = 50 μm

HMGB1 translocation and release play important roles in TBI-induced microglial activation and the subsequent inflammatory response [15, 37]. Using double immunofluorescent staining, HMGB1 expressions in neurons (NeuN+), microglia (Iba-1+), and astrocytes (GFAP+) were assessed in lesioned cortices 3 days after TBI**.** Compared with the sham group, the expression levels of HMGB1 were higher in the TBI group 3 days after injury. After ω-3 PUFA supplementation, HMGB1 expression was inhibited in both neurons and microglia, but not in astrocytes (Fig. 4a–c).

**Fig. 5:**
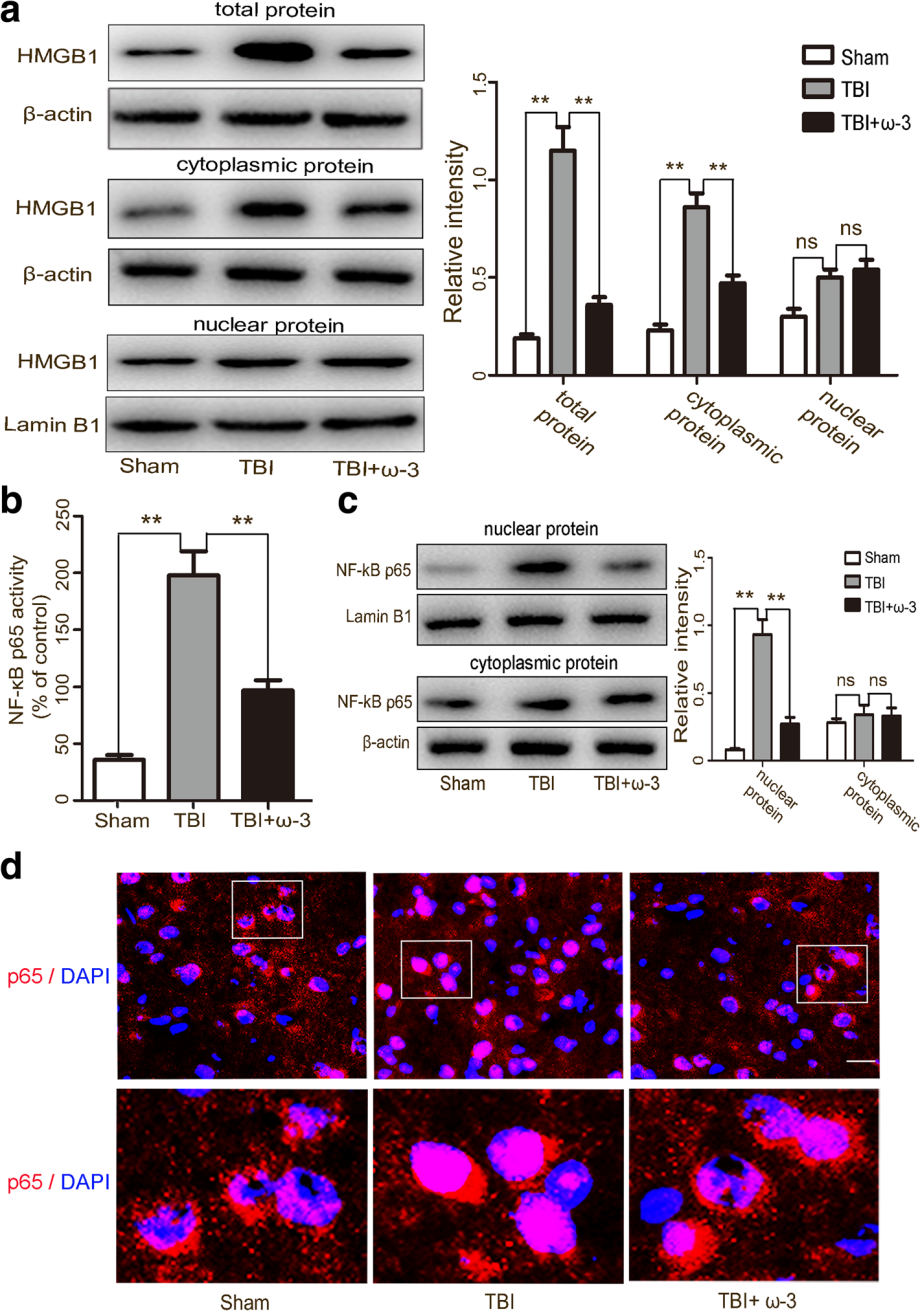
ω-3 PUFA supplementation inhibits the HMGB1/ NF-κB pathway in lesioned cortices 3 days after TBI. **a** Western blot analysis demonstrated that expression levels of HMGB1 in the cytosol, nuclei and in total protein of neurons and microglias from lesioned cortices increased after TBI. ω-3 PUFA supplementation significantly decreased HMGB1 expression in cytosolic and total cellular protein levels (*p* < 0.05), but not nuclearprotein (*p* > 0.05). **b** A significant reduction in NF-κB p65 DNA binding activity was observed in the TBI + ω-3 group compared with the TBI group (*p* < 0.05). **c** ω-3 PUFA supplementation significantly inhibited the translocation of NF-κB p65 from the cytosol to the nucleus and reduced NF-κB p65 expression (*p* < 0.05). ω-3 PUFA supplementation inhibited NF-κB p65 translocation to the nucleus (*p* < 0.05). **d** Representative photomicrographs of NF-κB p65 staining in the experimental groups. Values are expressed as mean ± standard deviation (*n* = 6 per group). N.S., *p* > 0.05, *p < 0.05, **p < 0.01. Scale bars = 50 μm

Western blot analyses demonstrated that expression levels of HMGB1 in the cytosol, nuclei, and in total protein of neurons and microglias from lesioned cortices increased after TBI, but ω-3 PUFA supplementation effectively decreased HMGB1 expression in the cytosol and in total protein of cells from lesioned cortices (*p* < 0.05), but not in nuclearprotein (*p* > 0.05) (Fig. 5a). Activation of the NF-κB pathway is associated with HMGB1 expression and the post-TBI inflammatory response [4]. A significant reduction in NF-κB p65 DNA binding activity was observed in the TBI + ω-3 group compared with the TBI group (*p* < 0.05) (Fig. 5b). Western blot and immunofluorescence staining further showed that compared with the TBI group, ω-3 PUFA supplementation significantly inhibited the translocation of NF-κB p65 from the cytosol to the nucleus, reduced NF-κB p65 expression, and attenuated NF-κB p65 DNA-binding activity (Fig. 5c, d). These results indicate that ω-3 PUFA supplementation can inhibit HMGB1 expression and extracellular secretion in both neurons and microglias, thus inhibiting the NF-κB pathway and attenuating the inflammatory response after TBI.

### ω-3 PUFA supplementation elevates SIRT1 expression and deacetylase activity

**Fig. 6:**
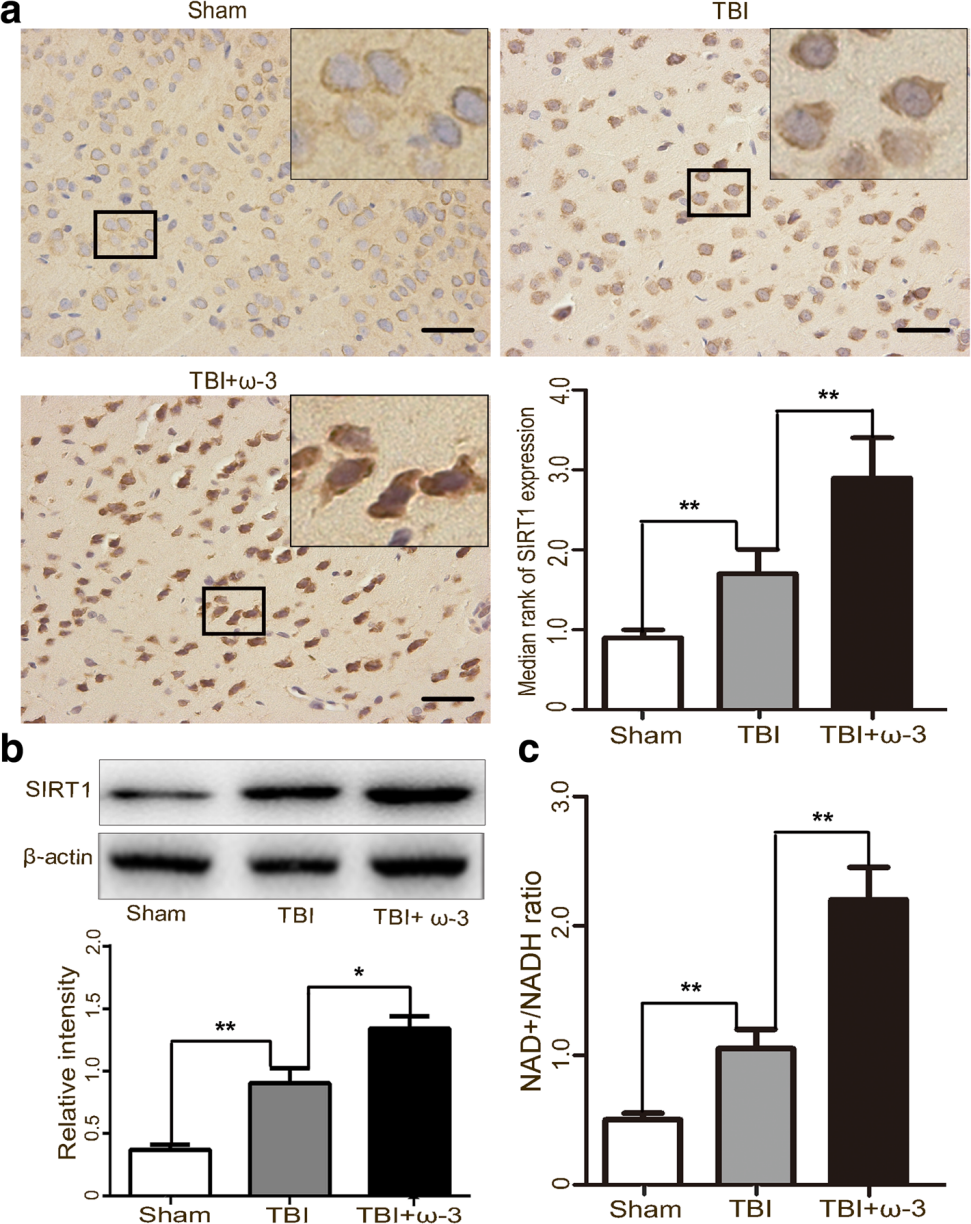
ω-3 PUFA supplementation elevates SIRT1 expression and deacetylase activity in lesioned cortices 3 days after TBI. **a** SIRT1 immunoreactivity in both neurons and microglias from lesioned cortices was significantly increased by ω-3 PUFA supplementation (2.92 ± 0.52 vs. 1.86 ± 0.29, *p* < 0.05). **b** SIRT1 levels were also upregulated after ω-3 PUFA supplementation (*p* < 0.05). **c** The NAD+/NADH ratio was measured to detect SIRT1 activity. Treatment with ω-3 PUFA significantly increased the NAD+/NADH ratio (*p* < 0.05). Values are expressed as mean ± standard deviation (*n* = 6 per group). N.S., *p* > 0.05, *p < 0.05, **p < 0.01. Scale bars = 50 μm

SIRTs are a family of deacetylases that require nicotinamide adenine dinucleotide (NAD+) as a cofactor for the deacetylation reaction [22, 23]. Consistent with our previous study, similar results were obtained by immunohistochemistry: SIRT1 immunoreactivity in both neurons and microglias from lesioned cortices was significantly increased after ω-3 PUFA supplementation (2.92 ± 0.52 vs. 1.86 ± 0.29, *p* < 0.05) (Fig. 6a). SIRT1 protein levels were also upregulated after ω-3 PUFA supplementation (*p* < 0.05) (Fig. 6b). As SIRT1 is a NAD+-dependent histone deacetylase that affects NAD+ metabolism [24, 38], we also measured the NAD+/NADH ratio to detect SIRT1 activity. Treatment with ω-3 PUFA significantly increased the NAD+/NADH ratio (*p* < 0.05) (Fig. 6c).

### ω-3 PUFA supplementation suppresses the HMGB1 pathway by elevating SIRT1 activity

**Fig. 7:**
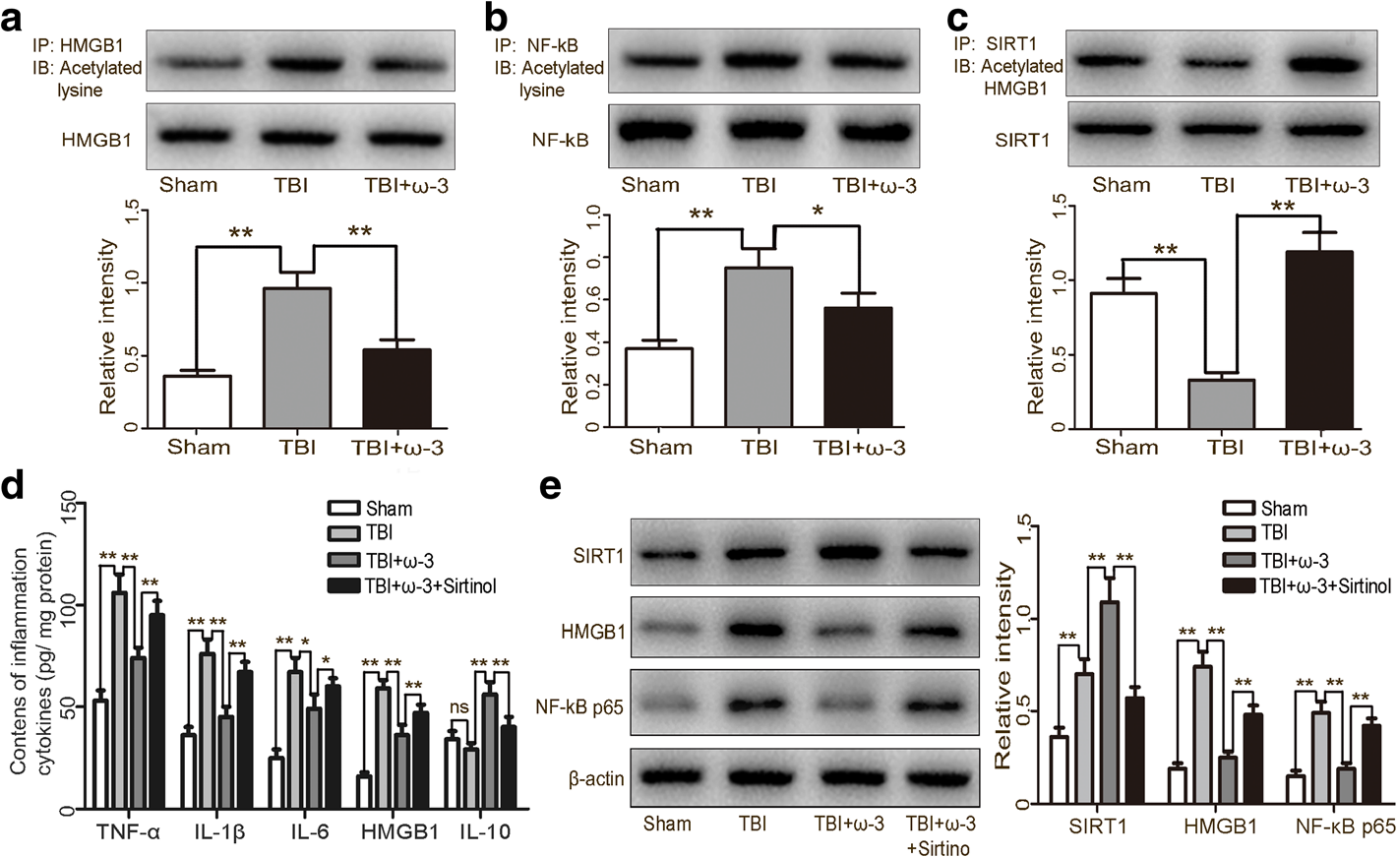
ω-3 PUFA supplementation suppressed HMGB1 pathway via elevating SIRT1 activity in lesioned cortices 3 days after TBI. **a** Co-IP analysis showed that a reduction in HMGB1 acetylation following ω-3 PUFA supplementation compared with the TBI group (*p* < 0.05). **b** Co-IP analysis showed a reduction in NF-κB acetylation following ω-3 PUFA supplementation compared with the TBI group (*p* < 0.05). **c** HMGB1 acetylation caused it to dissociate from SIRT1, thereby promoting HMGB1 extracellular secretion after TBI; ω-3 PUFA supplementation also induced direct interactions between SIRT1 and HMGB1 (*p* < 0.05). **d** The inhibitory effect of ω-3 PUFA supplementation on the neuroinflammatory response was reversed by pharmacological inhibition of SIRT1. **e** The inhibitory effect of ω-3 PUFA supplementation on HMGB1/NF-κB pathway activation was reversed by pharmacological inhibition of SIRT1. Values are expressed as mean ± standard deviation (*n* = 6 per group). N.S., *p* > 0.05, *p < 0.05, **p < 0.01. Scale bars = 50 μm

Posttranslational modifications such as acetylation are critical for HMGB1 transcription and extracellular secretion [21]. SIRT1 inhibits HMGB1 transcription and extracellular secretion by keeping HMGB1 in a deacetylated (inactive) state which sequesters the inflammatory response [21, 26]. Co-immunoprecipitation (Co-IP) analysis showed that both HMGB1 and NF-κB acetylation were decreased in acetyl-lysine immunoprecipitate fractions after ω-3 PUFA supplementation compared with the TBI group (*p* < 0.05) (Fig. 7a, b). To verify whether HMGB1 acetylation formed a direct complex with SIRT1, we assessed the impact of SIRT1 deacetylase activity on the interaction between HMGB1 and SIRT1. Co-IP analysis further showed that the acetylation of HMGB1 caused it to dissociate from SIRT1, thereby promoting HMGB1 extracellular secretion after TBI; ω-3 PUFA supplementation induced direct interactions between SIRT1 and HMGB1 (*p* < 0.05) (Fig. 7c). The inhibitory effects of ω-3 PUFA supplementation on the inflammatory response and on SIRT1-HMGB1/NF-κB axis activation were reversed by pharmacological inhibition of SIRT1, suggesting that the anti-inflammatory effect of ω-3 PUFA was dependent on SIRT1 activity (Fig. 7d, e).

## Discussion

**Fig. 8:**
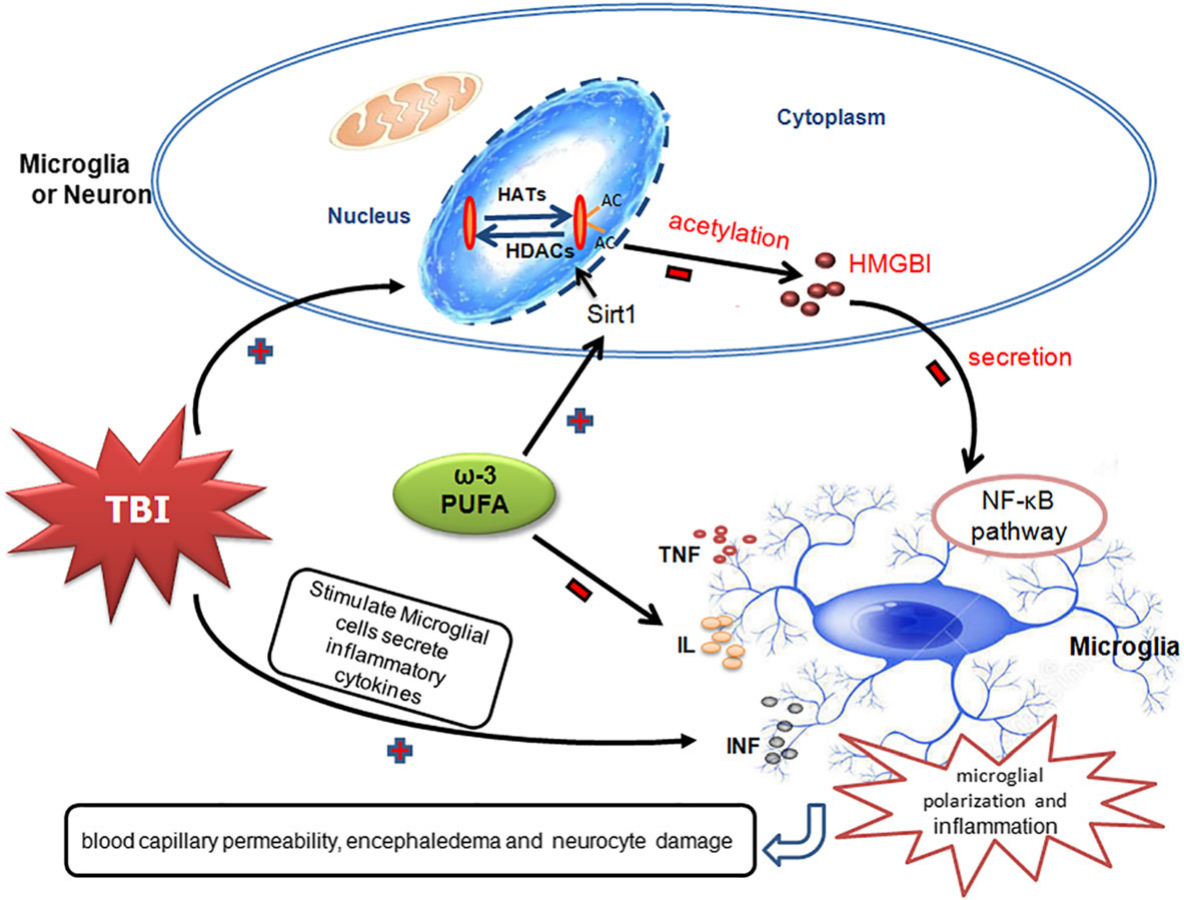
Schematic illustration of the possible neuroprotective mechanisms of ω-3 PUFA supplementation after TBI. As illustrated, TBI-induced microglial activation initiates a neuronal-glial neuroinflammatory response by producing a wide array of pro-inflammatory factors or mediators such as TNF, ILs, and IFN. Under inflammatory conditions, activation of the HMGB1/NF-κB pathway is closely related to its acetylation levels, which is regulated by SIRT1. Supplementation with ω-3PUFA attenuates the inflammatory response by modulating microglial polarization through SIRT1-mediated deacetylation of HMGB1/NF-κB pathway, leading to neuroprotective effects following experimental traumatic brain injury

Accumulating evidence has demonstrated the benefits of ω-3 PUFA or its constituents against TBI-induced neural damage and secondary pathological processes [32–34]. We previously reported that ω-3 PUFA supplementation inhibited TBI-induced microglial activation and the subsequent inflammatory response, leading to neuroprotective effects [4]. Taken together with our previously reported findings, the present study supports the view that ω-3 PUFA is a suitable therapeutic candidate against trauma-induced mechanical injury and secondary neuronal apotosis. Furthermore, ω-3 PUFA supplementation reduced brain edema and BBB permeability, and improved neurological function in lesioned cortices by inhibiting the expression of the pro-apoptotic factors, cleaved caspase-3 and Bax, and increasing expression of the anti-apoptotic factor, Bcl-2 (Fig. 8).

Microglial activation and the subsequent neuroinflammatory response were reported to be associated with decreased mitochondrial membrane potential and increased release of cytochrome C. Caspase activity contributes to neuronal apoptosis [39, 40]. Apoptotic event can cause further damage within the brain through the process of microglial activation, which is a vital contributing factor of TBI-induced secondary injury [1–3]. A transition from an anti-inflammatory M2 phenotype to a pro-inflammatory M1 phenotype plays a crucial role in microglial activation and the resulting neuroinflammatory response [1, 2, 7]. Our study showed that activation of microglia and expression of inflammatory factors (TNF-α, IL-1β, IL-6, and HMGB1) were significantly enhanced in brain tissue after TBI, which were associated with brain edema and neurological deficits. In addition, ω-3 PUFA supplementation promoted a shift from the M1 to the M2 phenotype and inhibited microglial activation, thus reducing TBI-induced inflammatory factors. These findings were consistent with the observation that ω-3 PUFA supplementation reduced neuronal apoptosis following TBI. Our results suggest that ω-3 PUFA supplementation may modulate microglial polarization, decrease microglial activation and the subsequent inflammatory response, reduce brain edema, inhibit apoptosis, and improve neurological functions.

HMGB1 translocation, release, and activation of the NF-κB signaling pathway are considered to be pivotal in the TBI-induced inflammatory response due to the secretion of pro-inflammatory factors [41, 42]. An increase in HMGB1 nucleocytoplasmic translocation and extracellular secretion, and the activation of HMGB1/NF-κB, coupled with increased expression of pro-inflammatory factors, were detected after TBI [4]. Furthermore, clinical evidence suggests that elevation of HMGB1 in cerebrospinal fluid was correlated with neurologic outcome in TBI patients [43]. Measuring HMGB1 might represent a potentially predictive biomarker in the early detection of post-trauma complications, suggesting inhibition of HMGB1 and extracellular secretion might offer a novel anti-inflammatory strategy to protect against TBI [43]. Our previous study [4] confirmed that inhibition of HMGB1 translocation release and activation of the NF-κB signaling pathway led to a reduction in TBI-induced microglial activation and the subsequent inflammatory response, thus providing neuroprotection following TBI. Consistent with our previous findings [4], we showed that expression levels of HMGB1 in both neurons and microglia were higher in the TBI group 3 days after injury. We also found that after ω-3 PUFA supplementation, HMGB1 expression was inhibited in both neurons and microglia in lesioned cortices. Moreover, ω-3 PUFA supplementation inhibited HMGB1 translocation, release, and NF-κB pathway activation after TBI. These results suggest that HMGB1 has important roles in microglial polarization and the neuroinflammatory response after TBI.

Activation of the HMGB1/NF-κB pathway is closely related to HMGB1 acetylation, which is regulated by the balance of HDACs and HATs [19, 20]; furthermore, HMGB1 mediates late inflammatory responses, which are related to protein acetylation levels [12, 44]. HMGB1 acetylation was previously found to promote the cytoplasmic accumulation and secretion of lysosomes [19, 20, 45]. SIRTs are a family of deacetylases that require NAD+ as a cofactor for the deacetylation reaction [22]. Our previous study [4] confirmed that the expression of acetylated HMGB1 and SIRT1 activity were involved in inflammatory mechanisms after TBI. In addition, we also found that SIRT1 levels were upregulated after ω-3 PUFA supplementation, indicating that ω-3 PUFA inhibited the expression and release of HMGB1 in a SIRT1 deacetylation-mediated dependent manner. Despite an increasing number of studies [24–26] showing that SIRT1 promotes the deacetylation of HMGB1, thus inhibiting HMGB1 transcription and extracellular release, the anti-inflammatory effect of the SIRT1-HMGB1 axis underlying TBI remains relatively under explored.

As SIRT1 is an NAD+-dependent histone deacetylase that affects the NAD+ metabolism, the NAD+/NADH ratio was measured to detect SIRT1 activity [24, 38]. We showed that treatment with ω-3 PUFA significantly increased the NAD+/NADH ratio and SIRT1 activity following TBI. Posttranslational modifications such as acetylation are critical for HMGB1 transcription and its extracellular secretion [21]. SIRT1 was also shown to inhibit HMGB1 transcription and extracellular secretion by maintaining HMGB1 in a deacetylated state, hence repressing the inflammatory response [21, 26]. Here, we found a novel counter-regulatory relationship between the attenuation of TBI-mediated HMGB1/NF-kB p65 pathway activity and regulation of SIRT1 overexpression. Our co-IP analysis also showed the presence of HMGB1 in acetyl-lysine immunoprecipitate fractions, confirming a decrease in HMGB1 acetylation after ω-3 PUFA supplementation. To verify whether acetylated HMGB1 formed a direct complex with SIRT1, we assessed the impact of SIRT1 deacetylase activity on the interaction between HMGB1 and SIRT1. Results showed that HMGB1 acetylation caused it to dissociate from SIRT1, thereby promoting HMGB1 extracellular secretion after TBI. Furthermore, ω-3 PUFA supplementation not only increased SIRT1 expression and HMGB1 deacetylation but also induced direct interactions between the two. The downregulation of acetylated HMGB1 promoted the formation of a complex with nuclear SIRT1, thereby inhibiting HMGB1 release and attenuating the central inflammatory response following TBI. These results indicate that ω-3 PUFA-mediated downregulation of HMGB1 acetylation and its complex with SIRT1 were likely due to an upregulation of SIRT1 (Fig. 8).

Activation of the NF-κB pathway is associated with HMGB1 expression and the post-TBI inflammatory response [17, 18]. Treatment with ω-3 PUFA significantly inhibited the translocation of NF-κB p65 from the cytosol to the nucleus, reduced NF-κB p65 acetylation, and attenuated NF-κB p65 DNA-binding activity, thus modulating downstream inflammatory responses. Sirtinol has many off-target effects, as there are iron chelation and biological effects below the SIRT protein inhibition levels. However, as a pharmacological inhibition of SIRT1, Sirtinol effectively inhibited the expressions of SIRT1 and SIRT2 and was used to inhibit SIRT1 signaling in several studies [22, 46, 47]. In our study, we also showed intervention with SIRT1 significantly decreased the SIRT1 expression following TBI. Moreover, the inhibitory effects of ω-3 PUFA supplementation on the inflammatory response and on SIRT1-HMGB1/NF-κB axis activation were reversed by Sirtinol, suggesting the anti-inflammation effects of ω-3 PUFAs were SIRT1 dependent. Future studies involving HMGB1 knockout mice are warranted to further investigate the mechanisms involved in ω-3 PUFA-mediated inhibition of HMGB1 and subsequent activation of the NF-κB pathway. Meanwhile, additional in vitro experiments are also needed to confirm the direct effects of ω-3 PUFA supplementation on neuronal and microglial activation.

## Conclusions

In summary, ω-3 PUFA supplementation promoted a shift of microglia from the M1 to the M2phenotype, inhibited microglial activation, and thus reduced TBI-induced inflammatory factors. In addition, ω-3 PUFA-mediated downregulation of HMGB1 acetylation and its extracellular secretion were likely due to increased SIRT1 activity. This indicates that treatment with ω-3 PUFA inhibited HMGB1 acetylation and induced direct interactions between SIRT1 and HMGB1 by increasing SIRT1 activity following TBI. These interactions then led to inhibition of HMGB1 nucleocytoplasmic translocation/extracellular secretion, which sequestered HMGB1-mediated activation of the NF-κB signaling pathway following TBI-induced microglial activation and thus inhibited the subsequent inflammatory response (Fig. 8).

## Abbreviations

BBB: Blood-brain barrier
co-IP: Co-immunoprecipitation
DHA: Docosahexaenoic acid
ELISA: Enzyme-linked immunosorbent assay
EPA: Eicosapentaenoic acid
HATs: Histone acetyltransferases
HDACs: Histone deacetylases
HMGB1: High mobile group box 1
IFN: Interferon
IL: Interleukin
MAPK: Mitogen-activated protein kinase
mNSS: Modified neurological severity scores
NAD: Nicotinamide adenine dinucleotide
NF-κB: Nuclear factor-κB
PI3K: Phosphatidylinositol 3-kinase
RAGE: Glycation end products
SIRT: Sirtuin
TBI: Traumatic brain injury
TLRs: Toll-like receptors
TNF: Tumor necrosis factor
TUNEL: Terminal deoxynucleotidyl transferase dUTP nick-end labelling
ω-3 PUFA: Omega-3 polyunsaturated fatty acid
